# Ancient DNA of northern China Hystricidae sub-fossils reveals the evolutionary history of old world porcupines in the Late Pleistocene

**DOI:** 10.1186/s12862-020-01656-x

**Published:** 2020-07-18

**Authors:** Guilian Sheng, Jiaming Hu, Haowen Tong, Bastien Llamas, Junxia Yuan, Xindong Hou, Shungang Chen, Bo Xiao, Xulong Lai

**Affiliations:** 1 School of Environmental Studies, China University of Geosciences, Wuhan, 430078 China; 2 State Key Laboratory of Biogeology and Environmental Geology, China University of Geosciences, Wuhan, 430078 China; 3 Key Laboratory of Vertebrate Evolution and Human Origins of Chinese Academy of Sciences, Institute of Vertebrate Paleontology and Paleoanthropology, Chinese Academy of Sciences, Beijing, 100044 China; 4 Australian Centre for Ancient DNA, School of Biological Sciences, Environment Institute, University of Adelaide, Adelaide, SA 5000 Australia; 5 Faculty of Materials Science and Chemistry, China University of Geosciences, Wuhan, 430074 China; 6 Faculty of Earth Sciences, China University of Geosciences, Wuhan, 430074 China

**Keywords:** Porcupine, Ancient DNA, Phylogenetic analysis, Evolutionary history, Pleistocene, China

## Abstract

**Background:**

Old World porcupines (Family: Hystricidae) are the third-largest rodents and inhabit southern Europe, Asia, and most regions of Africa. They are a typical indicator of warm climate and their distribution is restricted to tropical and subtropical zones. In China, porcupines are widely distributed in southern areas of the Yangtze River. However, fossil remains have been identified in a few sites in northern China, among which Tianyuan Cave—near Zhoukoudian site—represents the latest known porcupine fossil record. So far, studies have focused mainly on porcupines’ husbandry and domestication but little is known about their intrafamilial phylogenetic relationships and evolutionary history.

**Results:**

In this study, we sequence partial mitochondrial 12S rRNA and *cyt b* genes for seven Late Pleistocene porcupine individuals from Northern, Southern and Central China. Phylogenetic analyses show that the Tianyuan Cave porcupines, which had been morphologically identified as *Hystrix subcristata*, have a closer relationship to *Hystrix brachyura*.

**Conclusion:**

Together with morphological adaptation characteristics, associated fauna, and climate change evidence, the molecular results reveal that a Late Quaternary extirpation has occurred during the evolutionary history of porcupines.

## Background

Hystricidae (the Old World porcupines), which includes three distinguishable genera (*Hystrix*, *Atherurus*, *Trichys*), is one of the four families contained in the infraorder Phiomorpha [1, 2]. Phylogenetic evidence led to the hypothesis that the genera *Trichys* and *Atherurus* originated in the Paleocene from an unknown hystricognath ancestor in Asia [3]. However, the earliest porcupine fossils were recorded only in the Miocene in Europe [1]. The extant Hystricidae species are distributed in Asia, Africa and Europe (only in Italy) [4]. Based on their habitat environment, porcupines are generally adapted to tropical and subtropical climate.

**Fig. 1:**
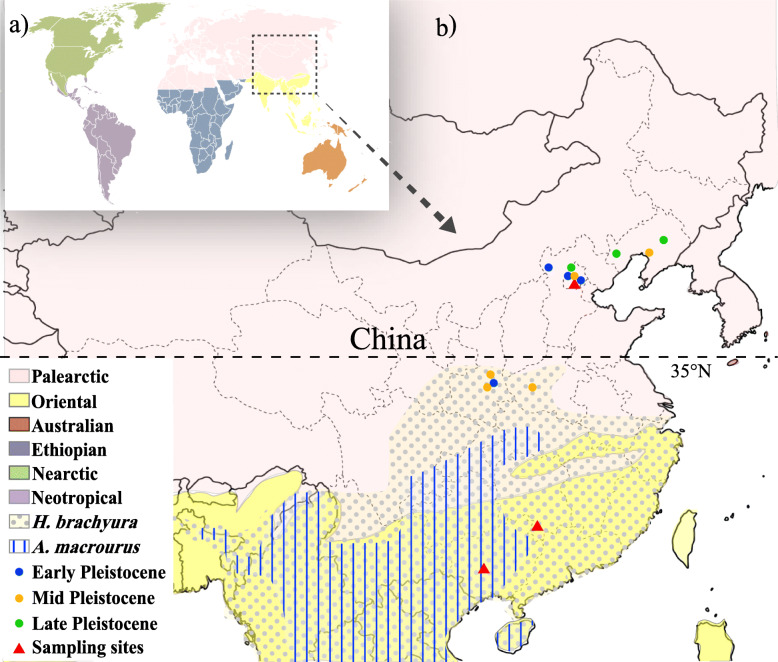
Sampling locations of porcupines investigated in this study. **a** Six global zoogeographic regions indicated by different color shadings; **b** Geographic distribution of the Hystricidae in Northern China during the Quaternary. The samples in this study are indicated by red triangles. Other Hystricidae fossils are represented by dots in three different colors [5]. Habitats of two extant porcupine species (*H. brachyura* and *A. macrourus*) are indicated by different patterns of shading [4]. The base map of global world (chart no. GS (2016)1611) and China (chart no. GS (2019)1694) were downloaded from the National Adminstration of Surveying, Mapping and Geoinformation of China (http://bzdt.ch.mnr.gov.cn)

In China, two species from two genera exist, i.e., *A. macrourus* (brush-tailed porcupine) and *H. brachyura* (Malayan porcupine) [4]. The latitude range of *H. brachyura* is 5°S–35°N, which totally encompasses the range of *A. macrourus,* i.e. 1°N–31°N (Fig. 1). A large number of *Hystrix* fossils have been excavated from the Chinese Quaternary strata. The oldest porcupine discovered in Asia, *H. lufengensis*, has been dated to the Late Miocene (8 Ma; million years ago) and was located in Yunnan Province, Southern China [6]. Fossils are particularly abundant in southern China compared to northern China, with fossil findings reported in nearly all provinces in subtropical areas from the Late Miocene to the Holocene. Nevertheless, typical porcupine fossils are present in northern China throughout the whole Quaternary [5]. Among all northern excavation sites, Zhoukoudian, Beijing has the richest assemblages of northern porcupine fossils and represents the latest known record before *Hystrix* finally disappeared from the Beijing area [5].

Morphologically, Quaternary porcupine fossils are quite distinct from extant species [7]. Clarifying the evolutionary history of porcupines is of great significance to better understand their response to climate change during their geographic extirpation in northern China during the Quaternary. However, molecular phylogenetic relationships between fossils and extant porcupines remain unclear. In this study, we extracted ancient DNA and sequenced partial cytochrome b (*cyt b*) and 12S ribosomal ribonucletic acid (rRNA) genes from Late Pleistocene Hystricidae fossils/sub-fossils in China. We constructed a phylogenetic tree to identify the evolutionary status of these fossils/sub-fossils and further discussed mechanisms underlying the range contraction of porcupines.

## Results

### Sequence recovery

Via multiple DNA (deoxyribonucleic acid) extractions, multiple amplifications, cloning of amplification products, sequencing of multiple clones, and replication of results by an independent laboratory, we analyzed seven Late Pleistocene fossil and sub-fossil porcupines: two from Tianyuan Cave, Zhoukoudian, Beijing (Specimens Nos.: CADG-67, CADG-68; 30.5–4.67 kiloannus (Ka)); two from Fuyan Cave, Daoxian County in Hunan province (Specimens Nos.: CADG-69, CADG-70; 153.8–129.6 Ka), and three from Loushan Cave, Wuming County in Guangxi province (Specimens Nos.: CADG-71, CADG-72, CADG-73; 24–13 Ka), respectively (Figs. 1 and S1). Seven overlapping fragments of the mitochondrial *cyt b* gene ranging from 90 to 111 base pairs (bp) (excluding primers) were amplified and sequenced for the two specimens from Tianyuan Cave at Zhoukoudian in Beijing (525 bp total for CADG-67, GenBank Accession No: MK579190; 605 bp for CADG-68, GenBank Accession No: MK579191). Another five overlapping fragments of the 12S rRNA gene ranging from 66 to 186 bp (excluding primers) were amplified and sequenced for the same samples (601 bp for CADG-67, GenBank Accession No: MK419154; 573 bp for CADG-68, GenBank Accession No: MK419155). Identical sequences were obtained when experiments were independently replicated in the State Key Laboratory for Biogeology and Environmental Geology at the China University of Geosciences (Wuhan) and Australian Centre for Ancient DNA at The University of Adelaide. In contrast, no fragments could be recovered from five samples collected from Hunan Province and Guangxi Province despite repeated attempts. No sequences were successfully amplified from all extraction and PCR (polymerase chain reaction) blank controls. Eventually, we considered only Tianyuan Cave samples for further phylogenetic analyses.

### Phylogenetic relationships

To investigate the phylogenetic position of the fossil porcupines in the Hystricidae family, we downloaded various sequences from GenBank and composed three datasets to use the available sequences as much as it is possible (Table S3).

**Fig. 2:**
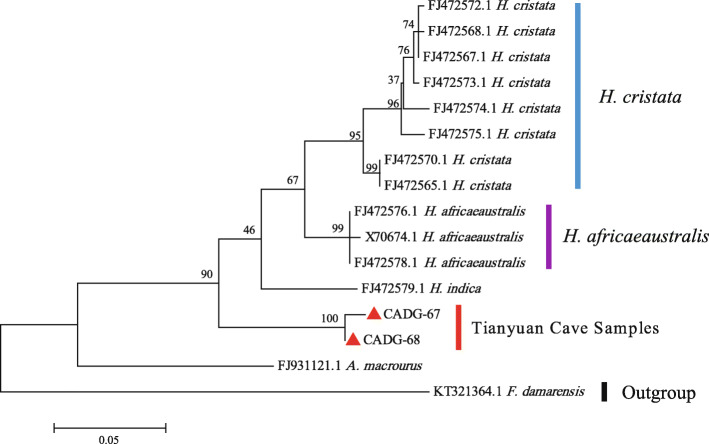
Phylogenetic Maximum Likelihood tree of species within Hystricidae using 446 bp of the *cyt b* gene. Tianyuan Cave samples are indicated by red triangles and bootstrap values are next to respective nodes

The relationship between Tianyuan Cave porcupines, *Hystrix* and *Atherurus* was inferred using Dataset 1 (446 bp of *cyt b* gene) in a Maximum Likelihood (ML) tree. Tianyuan Cave porcupines form a monophyletic clade basal to all *H. cristata* and *H. africaeaustralis*. *A. macrourus* is sister to all *Hystrix* and Tianyuan Cave samples (Fig. 2). Branch lengths suggest that the Late Pleistocene Chinese porcupines are more related to *Hystrix* than to *Atherurus*.

**Fig. 3:**
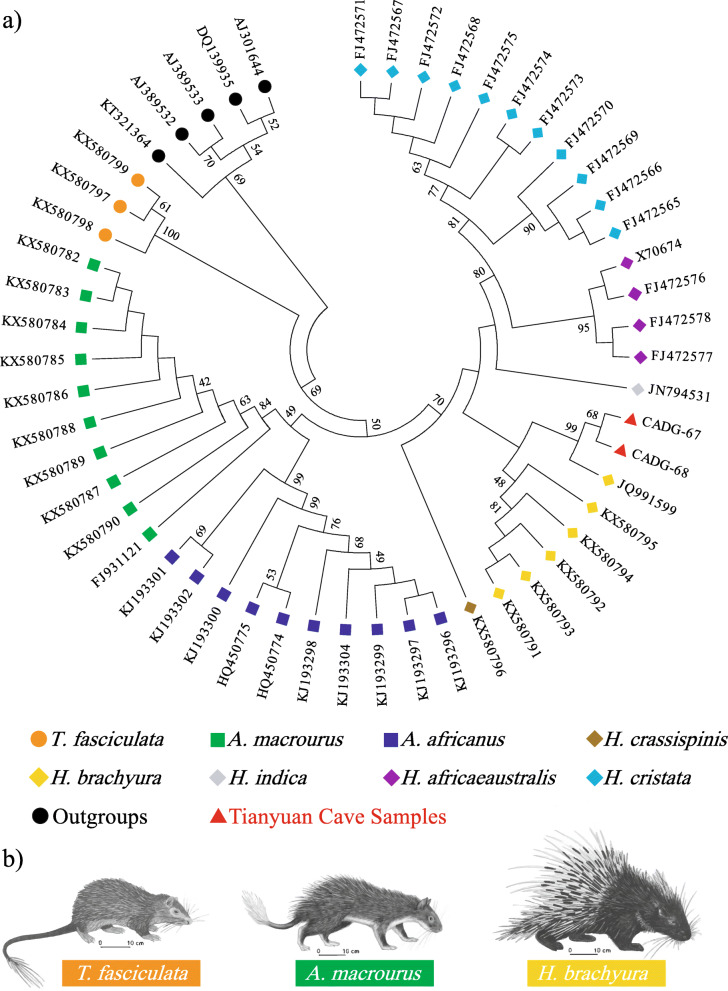
Molecular relationships of different species in the Hystricidae family shown by the 204 bp *cyt b* dataset and morphological characteristics of representative species. **a** Maximum Likelihood phylogenetic tree of Hystricidae. Tianyuan Cave samples are indicated by red triangles and bootstrap values are next to respective nodes. Only branches with bootstrap support greater than 40% are shown. **b** Representative species of three genera in Hystricidae [8, 9]

The molecular phylogeny of all species in the Hystricidae family with molecular data available was reconstructed using Dataset 2 (204 bp of *cyt b* gene) in a ML analysis. Individuals from three genera, i.e., *Atherurus*, *Hystrix*, and *Trichys* (Fig. 3b), form distinct groups, with *Trichys* standing basal in the tree. The two Late Pleistocene Chinese porcupines fall within the *Hystrix* group and are closely related to one *H. brachyura* individual from Thailand (GenBank Accession No: JQ991599). Although the relationship between *H. brachyura* from Peninsular Malaysia or Borneo and *H. indica* from India is generally poorly resolved, we can confidently reject the hypothesis that the Tianyuan Cave porcupines are either *H. cristata* or *H. africaeaustralis* (Fig. 3a).

**Fig. 4:**
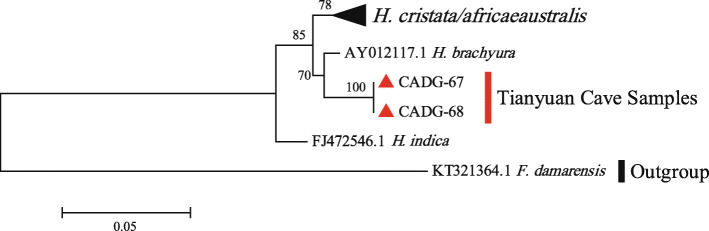
Phylogenetic Maximum Likelihood tree of species within Hystricidae based on 463 bp of the 12S rRNA gene. Tianyuan Cave samples are indicated by red triangles and bootstrap values are next to respective nodes. A monophyletic clade that comprises most *H. cristata/africaeaustralis* individuals is collapsed for simplicity (black triangle)

The molecular phylogeny based on 12S rRNA using Dataset 3 in a ML analysis is shown in Fig. 4. Results are consistent with the phylogeny obtained from the partial *cyt b* gene data set, except for one *H. indica* individual (GenBank Accession No: FJ472546) that falls basal in the family Hystricidae. The Tianyuan Cave porcupines form a monophyletic group and have a well-supported sister relationship with a *H. brachyura* individual (bootstrap value of 70%).

## Discussion

### DNA preservation of Late Pleistocene porcupines in China

Hydrolysis, oxidation and microbial action are the main factors that directly cause DNA fragmentation and degradation [10]. Therefore, the preservation of ancient DNA molecules is not obviously related to age of samples, whereas temperature, pH, ionic strength and humic acid content of the burial environment directly affect the degradation rate of ancient DNA [11]. Chronologically, all porcupine samples in this study have similar ages and have been dated to the Late Quaternary. However, we only successfully amplified ancient DNA fragments from specimens collected from Tianyuan Cave, while no positive results were obtained from the other two caves despite multiple attempts. Sediments in Tianyuan Cave have been deposited since 42 Ka and experienced four stages that correspond to four different climatic conditions [12]. The layer of our porcupine samples was at the third Stage, a cold and dry stage from the late Pleistocene to the early Holocene. Although we have no accurate information to estimate the paleo-environment of the sampling sites in Hunan and Guangxi provinces, it is clear that samples in these two sites were discovered in wet soil. We assume that the warm and humid environments in Hunan and Guangxi provinces accelerated DNA decay to the point of DNA fragments being too short for efficient PCR amplification using our primer sets [13]. Alternatively, it is possible that PCR inhibitors present in the soil were co-extracted with DNA, resulting in failure to amplify DNA. Of note, partial and even complete ancient mitochondrial DNA sequences have been successfully retrieved from megafauna samples collected from southern China [14, 15]. It is possible that the small porcupine bones did not preserve DNA as efficiently as in large mammal bones. In contrast, Tianyuan Cave is located in northern China, where the general cold and dry climate conditions are likely to better preserve ancient DNA for PCR-based analyses.

### Phylogenetic position of Tianyuan cave porcupines

Previous morphological identification named Tianyuan Cave porcupines as *H. subcristata* [5, 16]. There are various opinions with regard to the classification of *Hystrix* in China. Huang et al. [17] sorted Chinese *Hystrix* into three species: *H. indica*, *H. hodgsoni*, and *H. brachyura*. Weers [18] further classified *H. brachyura* into two subspecies: *H. brachyura subcristata* and *H. brachyura yunnanensis*. Instead of the subspecies status, Tong restored the original classification status of *H. subcristata* into a species level, since differences in measurements of skull height and nasal length/occipito-nasal length of *H. subcristata* and *H. brachyura* are indeed obvious [5]. In this study, both *cyt b* and 12S rRNA gene analysis support that Tianyuan Cave porcupines have a closer relationship with *Hystrix* than with other genera in the family Hystricidae (Figs. 2 and 3). However, some poorly resolved nodes within the *Hystrix* clade (Fig. 3) lead to an ambiguous phylogenetic assignment of the Tianyuan Cave porcupines at the species level. We can say with confidence that the Tianyuan Cave porcupines are not *H. cristata* or *H. africaeaustralis*, and that they are likely more closely related to *H. brachyura*. Homologous sequences from morphologically distinct *H. brachyura subcristata*, *H. brachyura yunnanensis*, or *H. subcristata* individuals are not available yet. Therefore, it seems that the taxonomic assignment of the Late Pleistocene porcupines in Tianyuan Cave as a subspecies or a species remains an open question in terms of molecular evidence. Further paleogenomic evidences from more contemporary and fossil individuals may help to solve this taxonomic issue.

### Porcupines in Tianyuan cave in relation with paleoclimate change

Among the family Hystricidae, the oldest genera *Trichys* have the smallest body size and the most rat-like appearance [9] (Fig. 3b). This genus is distributed in tropical areas near the equator such as Indonesia and the Malay Peninsula. The evolutionarily younger genera *Atherurus* and *Hystrix* are distributed in higher northern latitudes such as in Central Asia and Southern Europe. Therefore, the contemporary Hystricidae members include species adapted to warm climate in both tropical and subtropical zones. The Tianyuan Cave porcupines were located at 39° N in terms of latitude, while the distribution range of wild *H. brachyura* is generally no more than 35° N [4, 17] (Fig. 1). Moreover, the Tianyuan Cave samples are more closely related to one individual from Thailand (GenBank Accession No: JQ991599) than to individuals from Malaysia (GenBank Accession Nos: KX580791–5) (Fig. 3a). The associated fauna discovered together with Tianyuan Cave porcupine samples, such as *Macaca mulatta*, *Ursus thibetanus*, *Arctonyx collaris*, *Paguma larvata*, *Panthera pardus,* etc., are all warmth-adapted species [5, 19]. These warmth-adapted species, together with the phylogenetic relationship revealed by this study, indicate that mid-Holocene extirpation events have occurred in the evolutionary history of porcupines.

Many paleontologists indicated that the skeletal characteristics of porcupine are related to their evolutionary status and habitat environment [5, 7]. Wang and Qiu [7] compared eight species in the genera *Hystrix* that are distributed from Indonesia to central China and suggested that *Hystrix* seems to have the following evolutionary tendencies: the rostrum heightens and the nasal and frontal bones enlarge as latitude increases. By comparing the nasal length and other morphological traits between *Hystrix* species, Tong discovered that the skull of *Hystrix* in the tropics is rounder, while those in northern areas have longer nasal length. For instance, the two extant *Hystrix* (*H. cristata* and *H. brachyura subcristata*), which have the longest nasal length, are distributed in relatively higher latitudes [5]. It seems that Tianyuan Cave porcupines did have morphological characteristics making them to be better adapted to live in northern China. Cold climate indicator such as the loess record suggests that frequent paleoclimate fluctuations occurred during the Late Pleistocene in China [20]. This can also be verified by the coincidence of the frequent and dramatic sea level rises and falls during the same period [21].

Therefore, we hypothesize that porcupines have migrated to northern China during a warm period between the Late Miocene and the Early Pleistocene, and morphologically adapted to the relatively colder climate during the Late Pleistocene. They may have become locally extinct in the north during the Mid Holocene while the southern populations survived and formed the contemporary population distribution.

## Conclusion

In this study, we successfully analysed partial *cyt b* and 12S rRNA gene sequences from Tianyuan Cave porcupines. Phylogenetic analyses support a possible close relationship between our Late Pleistocene samples and extant *H. brachyura*, which are distributed in tropical and subtropical areas of Southeast and South Asia. Together with morphological adaptation characteristics and climate change evidence, our molecular results reveal a northern extirpation of porcupines during the Holocene.

## Methods

### DNA extraction and amplification

DNA extraction and other pre-amplification procedures were performed in a dedicated ancient DNA laboratory at the State Key Laboratory for Biogeology and Environmental Geology, China University of Geosciences (Wuhan), which is physically isolated from other molecular biology research facilities. Approximately 200 mg of tooth powder was used for extraction using an optimized silica in solution method [22]. Extraction and PCR blank controls were carried out throughout all experiments to monitor potential DNA contamination. Based on the mitochondrial genome sequence of extant *H. cristata* (GenBank Accession Nos: FJ472565–79), we designed 12 primer pairs (Supplementary Materials, Tables S1 and S2; Fig. S2) using Primer 5.0 (Premier Biosoft Interpairs). We attempted to amplify a 644-bp-long fragment of the 12S rRNA gene using 5 overlapping primer pairs and a 721-bp-long fragment of the *cyt b* gene using 7 overlapping primer pairs (Supplementary Materials, Fig. S2). Primer pairs were split into two pools of non-overlapping fragments for PCR amplification. A total of two multiplex PCRs were set up in 25 μL reactions using 4 ng of DNA template and 5 μM primer mix, 0.25 mM dNTPs (deoxy-ribonucleoside triphosphate), 2.5 mM MgSO_4_, 1× buffer, 10 mg/μL RSA, 1 U Taq DNA Polymerase. PCR cycling conditions were as follows: initial denaturation at 94 °C for 2 min, followed by 50 cycles of 94 °C for 15 s, 53 °C for 30 s and 68 °C for 20 s, ending with a final extension for 10 min at 68 °C. Then a simplex PCR was carried out for each individual primer pair using the same conditions as described above [23], using a 1:20 dilution of the respective multiplex PCR product. Simplex PCR products were purified using the QIAquick Gel Extraction Kit (Qiagen, Hilden, Germany) and ligated into the pMD18-T vector (Takara, Tokyo, Japan) following the supplier’s instructions. Competent *Escherichia coli* DH5α cells were transformed by heat shock method. Positive clones selected from LB plates were screened by PCR with the M13 primer pair. At least 8 clones for each fragment, 4 from each of 2 independent primary amplifications, were sequenced at Nanjing Genscript Ltd. on an ABI 3700 sequencer. We called the majority allele for positions where we observed substitutions between clones of a single PCR product. For the specimens from Tianyuan Cave (Supplementary Materials, Fig. S1), amplification and sequencing of all fragments of both *cyt b* and 12S rRNA was independently replicated at the University of Adelaide’s Australian Centre for Ancient DNA (Supplementary Materials, Fig. S2).

### Alignment and phylogenetic analyses

Sequence alignments were performed using the “ClustalW” function in BioEdit 7.2.6.1 [24], and assemblies were manually checked. BLAST analysis [25] was used to check sequence similarity. The four newly determined DNA sequences were deposited in GenBank (Accession Nos: MK419154–5; MK579190–1). To investigate the phylogenetic position of the fossil porcupines in the Hystricidae family, we downloaded the following sequences from GenBank: **1)** 51 sequences of *cyt b* that include 20 from the *Atherurus* genus, 23 from *Hystrix*, 3 from *Trichys*, and 5 outgroups; and **2)** 21 sequences of 12S rRNA that include one *H. brachyura*, three *H. africaeaustralis*, 15 *H. cristata*, one *H. indica* and one outgroup. Three datasets were used in different analyses (Supplementary Materials, Table S3). According to the species distribution indicated by IUCN (International Union for Conservation of Nature) Redlist, we renamed several *H. cristata* from Namibia, Zambia and South Africa (GenBank Accession. No: FJ472576–8 & FJ472540–5) as *H. africaeaustralis* and renamed one Israeli *H. cristata* as *H. indica* (GenBank Accession No: FJ4725769 & FJ472546) (Supplementary Materials, Table S3).

Dataset 1 comprises 16 sequences: two Chinese sub-fossil porcupines, eight *H. cristata*, three *H. africaeaustralis*, one *H. indica*, one *A. macrourus*, and one outgroup for which 446-bp of *cyt b* were available. This dataset comprises the longest homologous sequences to have enough resolution to determine the relationship between the Tianyuan Cave porcupines (previously identified as *H. subcristata*) and *H. cristata*.

Dataset 2 comprises 53 sequences, 204-bp in length, where all downloaded *cyt b* sequences and two Tianyuan Cave samples were included. This dataset was used to identify the phylogenetic position of our ancient samples among the three genera in the Hystricidae family.

Dataset 3 comprises 23 12S rRNA sequences (463-bp): two Chinese Pleistocene porcupines, one *H. brachyura*, one *H. indica*, seven *H. africaeaustralis*, 11 *H. cristata*, and one outgroup. This dataset was used to further confirm the phylogenetic position of the ancient samples and alleviate spurious false results caused by short sequences from Dataset 2.

We used 5 methods implemented in MEGA7 [26], i.e., Neighbor Joining (NJ), Maximum Likelihood (ML), Minimum Evolution (ME), Maximum Parsimony (MP), and Unweighted Pair Group Method with Arithmetic Mean (UPGMA), to construct the phylogenetic trees. To assess the robustness of these methods, bootstrapping [27] with 500 replicates were conducted.

## Supplementary information

**Additional file 1: Figure S1.** Photos of two Tianyuan Cave samples. **Fig. S2** Schematic view of the primers designed for porcupines (arrows flanking dotted lines), amplicon sizes in base pairs (on top of plain lines) and structure of valid sequences (CADG labels) obtained from Tianyuan Cave samples. (a) Seven overlapping fragments designed for the *cyt b* gene. (b) Five overlapping fragments designed for the 12S rRNA gene. **Table S1.** PCR primers of 1140-bp *cyt b* gene for the Pleistocene porcupines. The characters ‘F’ and ‘R’ in the ‘site’ column refer to ‘forward primer’ and ‘reverse primer’, respectively. **Table S2.** PCR primers of 966-bp 12S rRNA gene for the Pleistocene porcupines. The characters ‘F’ and ‘R’ in the ‘site’ column refer to ‘forward primer’ and ‘reverse primer’, respectively. **Table S3.** Datasets used in this study (excluding ancient samples).

## Data Availability

The assembled porcupine *cyt b* and 12S rRNA sequences have been deposited in the GenBank database (http://www.ncbi.nih.gov/genbank) under the accession numbers MK579190–1, MK419154–5, respectively.

## Abbreviations

*cyt b*: Cytochrome b
rRNA: Ribosomal ribonucletic acid
DNA: Deoxyribonucleic acid
Ka: Kiloannus
bp: Base pair
PCR: Polymerase chain reaction
dNTP: Deoxy-ribonucleoside triphosphate
IUCN: International Union for Conservation of Nature
mg: Milligram
NJ: Neighbor Joining
ML: Maximum Likelihood
ME: Minimum Evolution
MP: Maximum Parsimony
UPGMA: Unweighted pair group method with arithmetic mean
