# Supplementary material for: Ancient DNA of northern China Hystricidae sub-fossils reveals the evolutionary history of old world porcupines in the Late Pleistocene
Source: BMC Evol Biol. 2020 Jul 18;20:88. doi: 10.1186/s12862-020-01656-x (PMC7368748; doi:10.1186/s12862-020-01656-x)
Supplement: Supplementary file 1 — Additional file 1: Figure S1. Photos of two Tianyuan Cave samples. Fig. S2 Schematic view of the primers designed for porcupines (arrows flanking dotted lines), amplicon sizes in base pairs (on top of plain lines) and structure of valid sequences (CADG labels) obtained from Tianyuan Cave samples. (a) Seven overlapping fragments designed for the cyt b gene. (b) Five overlapping fragments designed for the 12S rRNA gene. Table S1. PCR primers of 1140-bp cyt b gene for the Pleistocene porcupines. The characters ‘F’ and ‘R’ in the ‘site’ column refer to ‘forward primer’ and ‘reverse primer’, respectively. Table S2. PCR primers of 966-bp 12S rRNA gene for the Pleistocene porcupines. The characters ‘F’ and ‘R’ in the ‘site’ column refer to ‘forward primer’ and ‘reverse primer’, respectively. Table S3. Datasets used in this study (excluding ancient samples). [file 12862_2020_1656_MOESM1_ESM.docx]

**Ancient DNA of Northern China Hystricidae Sub-Fossils Reveals the Evolutionary History of Old World Porcupines in the Late** **Pleistocene**

Guilian Sheng^1,2*^, Jiaming Hu^1^, Haowen Tong^3^, Bastien Llamas^4^, Junxia Yuan^5^, Xindong Hou^1^, Shungang Chen^5^, Bo Xiao^1^, Xulong Lai^2,6*^

**Supplementary Materials
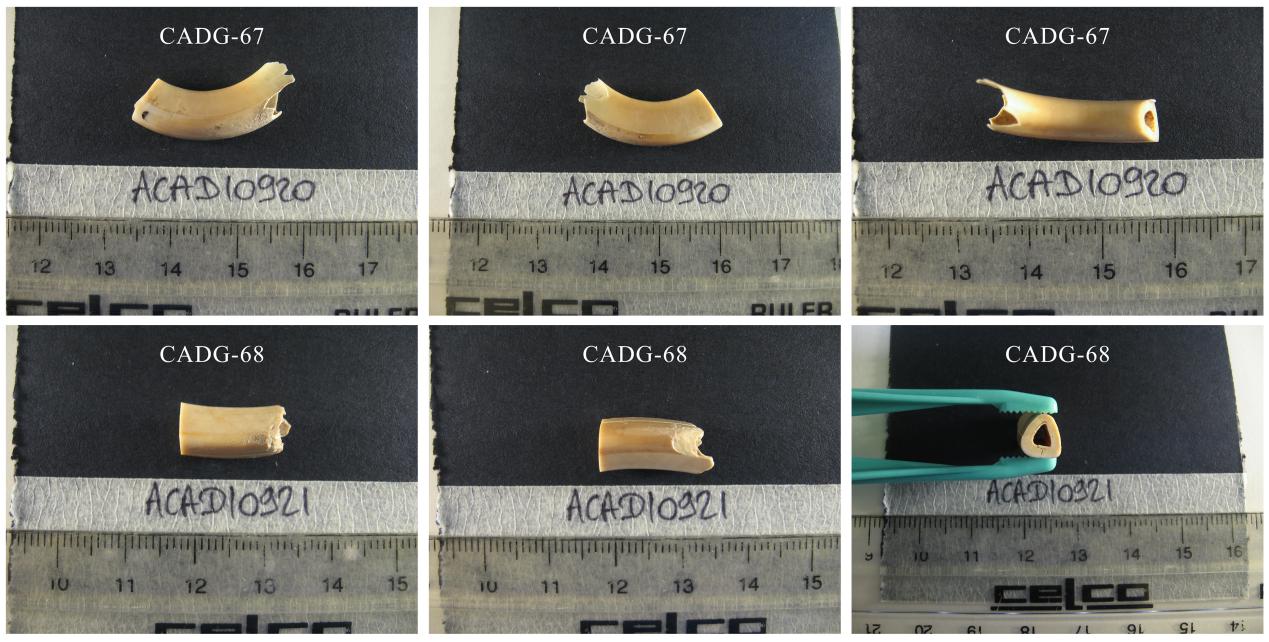
**

**Fig. S1** Photos of two Tianyuan Cave samples.


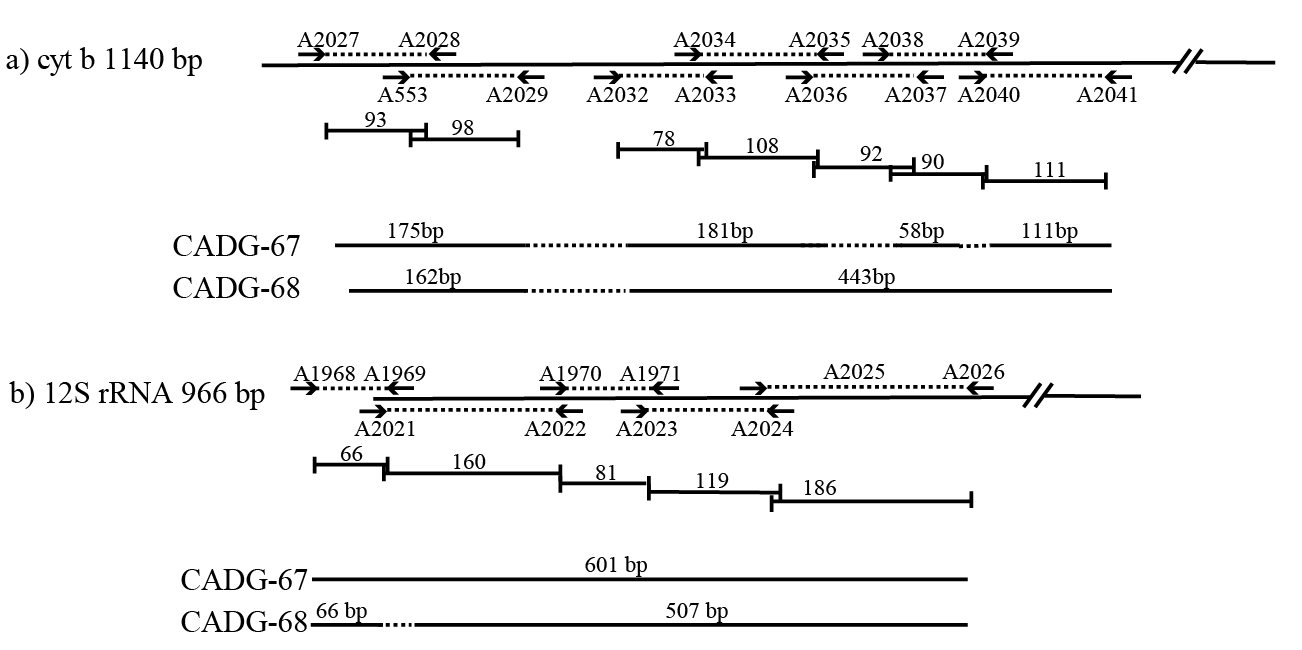


**Fig. S2** Schematic view of the primers designed for porcupines (arrows flanking dotted lines), amplicon sizes in base pairs (on top of plain lines) and structure of valid sequences (CADG labels) obtained from Tianyuan Cave samples. (a) Seven overlapping fragments designed for the *cyt b* gene. (b) Five overlapping fragments designed for the 12S rRNA gene.

**Table S1.** PCR primers of 1140-bp *cyt b* gene for the Pleistocene porcupines. The characters ‘F’ and ‘R’ in the ‘site’ column refer to ‘forward primer’ and ‘reverse primer’, respectively.

| Primer Name | Primers (5’ position to 3’ position) | Site | Amplicon Size (excluding primers) | Annealing Temperature（℃） |
| --- | --- | --- | --- | --- |
| A2027 | GAACTAATGACAAACATCCGAAAA | 24F | 114 bp | 50 |
| A2028 | GGATGTGGATGAGTAAGCAGGCTCC | 137R |  |  |
| A553 | CCCCCTCCAACATCTCCTCATGATGAAA | 88F | 127 bp | 50 |
| A2029 | CTCGGCAGATATGGGYTACTG | 214R |  |  |
| A2032 | CTGYCTATACCTCCACGTAGG | 276F | 120 bp | 50 |
| A2033 | GGACGTATCCTATGAARGCAGTAGC | 396R |  |  |
| A2034 | CGGACTTCTCTTACTGTTTACAG | 345F | 154 bp | 50 |
| A2035 | CCCTCAAATTCATGCAACTARTG | 498R |  |  |
| A2036 | CAGCAATCCCCTATCTTGGAAC | 452F | 135 bp | 50 |
| A2037 | TGGACTAGTACAAGGGCTGTGA | 586R |  |  |
| A2038 | AACACGATTCTTTGCTTTCCA | 525F | 132 bp | 50 |
| A2039 | GGAATTGTGTCTGAGTTTGRG | 656R |  |  |
| A2040 | GAAACAGGGTCAAACAACCCATC | 604F | 157 bp | 50 |
| A2041 | CTGAGTCTCCTACAAGGTCTAGG | 760R |  |  |

**Table S2.** PCR primers of 966-bp 12S rRNA gene for the Pleistocene porcupines. The characters ‘F’ and ‘R’ in the ‘site’ column refer to ‘forward primer’ and ‘reverse primer’, respectively.

| Primer Name | Primers (5’ position to 3’ position) | Site | Amplicon Size (excluding primers) | Annealing Temperature（℃） |
| --- | --- | --- | --- | --- |
| A1968 | GTCCATGTAGCTTAACACACA | 8F | 103 bp | 54 |
| A1969 | GTAACTAATGGAAAGGCTAGG | 110R |  |  |
| A1970 | TAGCCACATCCCCACGGAAATA | 244F | 136 bp | 54 |
| A1971 | GAGTTAATCGTATGACCGCGG | 379R |  |  |
| A2021 | CTCCATAGACATAAAGGTTTGG | 67F | 203 bp | 50 |
| A2022 | GCTGAATGGTGATCACTGCTG | 269R |  |  |
| A2023 | GGTAAATTTCGTGCCAGCC | 328F | 141 bp | 50 |
| A2024 | GAGAYTGCTTTCGCGTACTGG | 468R |  |  |
| A2025 | CGTACACCACTTCAGATTGATAC | 421F | 197 bp | 50 |
| A2026 | TATCGATTATAGAACAGGCTCC | 617R |  |  |

**Table S3.** Datasets used in this study (excluding ancient samples).

| No. | Species | Isolate | Location | GenBank Acc. No. | Dataset | Type |
| --- | --- | --- | --- | --- | --- | --- |
| 1 | *Atherurus africanus* | AafrT886_Niga | Nigeria | HQ450774 | 2 | *cyt b* |
| 2 | *Atherurus africanus* | AafrT966_Niga | Nigeria | HQ450775 | 2 | *cyt b* |
| 3 | *Atherurus africanus* | AafrBAY001 | Cameroon | KJ193296 | 2 | *cyt b* |
| 4 | *Atherurus africanus* | AafrCAM015 | Cameroon | KJ193297 | 2 | *cyt b* |
| 5 | *Atherurus africanus* | AafrCAM016 | Cameroon | KJ193298 | 2 | *cyt b* |
| 6 | *Atherurus africanus* | AafrCAM033 | Cameroon | KJ193299 | 2 | *cyt b* |
| 7 | *Atherurus africanus* | AafrCAMP001 | Cameroon | KJ193300 | 2 | *cyt b* |
| 8 | *Atherurus africanus* | AafrT1327 | Ghana | KJ193301 | 2 | *cyt b* |
| 9 | *Atherurus africanus* | AafrT1328 | Ghana | KJ193302 | 2 | *cyt b* |
| 10 | *Atherurus africanus* | AafrYAO002 | Cameroon | KJ193304 | 2 | *cyt b* |
| 11 | *Atherurus macrourus* | AM01 | Malaysia | KX580782 | 2 | *cyt b* |
| 12 | *Atherurus macrourus* | AM03 | Malaysia | KX580783 | 2 | *cyt b* |
| 13 | *Atherurus macrourus* | AM04 | Malaysia | KX580784 | 2 | *cyt b* |
| 14 | *Atherurus macrourus* | AM05 | Malaysia | KX580785 | 2 | *cyt b* |
| 15 | *Atherurus macrourus* | AM06 | Malaysia | KX580786 | 2 | *cyt b* |
| 16 | *Atherurus macrourus* | AM07 | Malaysia | KX580787 | 2 | *cyt b* |
| 17 | *Atherurus macrourus* | AM08 | Malaysia | KX580788 | 2 | *cyt b* |
| 18 | *Atherurus macrourus* | AM09 | Malaysia | KX580789 | 2 | *cyt b* |
| 19 | *Atherurus macrourus* | AM10 | Malaysia | KX580790 | 2 | *cyt b* |
| 20 | *Atherurus macrourus* | / | / | FJ931121 | 1&2 | *cyt b* |
| 21 | *Hystrix africaeaustralis* | / | / | X70674 | 1&2 | *cyt b* |
| 22 | *Hystrix brachyura* | HB03 | Malaysia | KX580791 | 2 | *cyt b* |
| 23 | *Hystrix brachyura* | HB05 | Malaysia | KX580792 | 2 | *cyt b* |
| 24 | *Hystrix brachyura* | HB06 | Malaysia | KX580793 | 2 | *cyt b* |
| 25 | *Hystrix brachyura* | HB07 | Malaysia | KX580794 | 2 | *cyt b* |
| 26 | *Hystrix brachyura* | HB109 | Malaysia | KX580795 | 2 | *cyt b* |
| 27 | *Hystrix brachyura* | Hbra01 | Thailand | JQ991599 | 2 | *cyt b* |
| 28 | *Hystrix cristata* | ITAA | Italy | FJ472565 | 1&2 | *cyt b* |
| 29 | *Hystrix cristata* | ITAA1 | Italy | FJ472566 | 2 | *cyt b* |
| 30 | *Hystrix cristata* | Tun02 | Tunisia | FJ472567 | 1&2 | *cyt b* |
| 31 | *Hystrix cristata* | Lib01 | Libya | FJ472568 | 1&2 | *cyt b* |
| 32 | *Hystrix cristata* | Mar01 | Morocco | FJ472569 | 2 | *cyt b* |
| 33 | *Hystrix cristata* | Mar02 | Morocco | FJ472570 | 1&2 | *cyt b* |
| 34 | *Hystrix cristata* | Eri01 | Eritrea | FJ472571 | 2 | *cyt b* |
| 35 | *Hystrix cristata* | Eri02 | Eritrea | FJ472572 | 1&2 | *cyt b* |
| 36 | *Hystrix cristata* | Eth01 | Ethiopia | FJ472573 | 1&2 | *cyt b* |
| 37 | *Hystrix cristata* | Tan01 | Tanzania | FJ472574 | 1&2 | *cyt b* |
| 38 | *Hystrix cristata* | BuF01 | Bukina Faso | FJ472575 | 1&2 | *cyt b* |
| 39 | *Hystrix africaeaustralis* | Nam02 | Namibia | FJ472576 | 1&2 | *cyt b* |
| 40 | *Hystrix africaeaustralis* | Nam03 | Namibia | FJ472577 | 2 | *cyt b* |
| 41 | *Hystrix africaeaustralis* | Nam05 | Namibia | FJ472578 | 1&2 | *cyt b* |
| 42 | *Hystrix indica* | In02 | Israel | FJ472579 | 1 | *cyt b* |
| 43 | *Hystrix indica* | KAU7810 | India | JN794531 | 2 | *cyt b* |
| 44 | *Hystrix crassispinis* | HBR01 | Malaysia | KX580796 | 2 | *cyt b* |
| 45 | *Trichys fasciculata* | TF01 | Malaysia | KX580797 | 2 | *cyt b* |
| 46 | *Trichys fasciculata* | TF02 | Malaysia | KX580798 | 2 | *cyt b* |
| 47 | *Trichys fasciculata* | TF03 | Malaysia | KX580799 | 2 | *cyt b* |
| 48 | *Ctenodactylus vali* | / | / | AJ389532 | 2 | *cyt b* |
| 49 | *Fukomys damarensis* | / | / | KT321364 | 1, 2 &3 | mitochondrion |
| 50 | *Massoutiera mzabi* | / | N/A | AJ389533 | 2 | *cyt b* |
| 51 | *Petromus typicus* | / | N/A | DQ139935 | 2 | *cyt b* |
| 52 | *Thryonomys swinderianus* | / | South Africa | AJ301644 | 2 | mitochondrion |
| 53 | *Hystrix brachyura* | / | / | AY012117 | 3 | 12S rRNA |
| 54 | *Hystrix cristata* | ITAA | Italy | FJ472530 | 3 | 12S rRNA |
| 55 | *Hystrix cristata* | Tun01 | Tunisia | FJ472531 | 3 | 12S rRNA |
| 56 | *Hystrix cristata* | Tun02 | Tunisia | FJ472532 | 3 | 12S rRNA |
| 57 | *Hystrix cristata* | Tun06 | Tunisia | FJ472533 | 3 | 12S rRNA |
| 58 | *Hystrix cristata* | Lib01 | Libya | FJ472534 | 3 | 12S rRNA |
| 59 | *Hystrix cristata* | Mar01 | Morocco | FJ472535 | 3 | 12S rRNA |
| 60 | *Hystrix cristata* | Mar03 | Morocco | FJ472536 | 3 | 12S rRNA |
| 61 | *Hystrix cristata* | Eri02 | Eritrea | FJ472537 | 3 | 12S rRNA |
| 62 | *Hystrix cristata* | Tan01 | Tanzania | FJ472538 | 3 | 12S rRNA |
| 63 | *Hystrix cristata* | BuF01 | Burkina Faso | FJ472539 | 3 | 12S rRNA |
| 64 | *Hystrix africaeaustralis* | Nam02 | Namibia | FJ472540 | 3 | 12S rRNA |
| 65 | *Hystrix africaeaustralis* | Nam05 | Namibia | FJ472541 | 3 | 12S rRNA |
| 66 | *Hystrix africaeaustralis* | Zam01 | Zambia | FJ472542 | 3 | 12S rRNA |
| 67 | *Hystrix africaeaustralis* | Zam02 | Zambia | FJ472543 | 3 | 12S rRNA |
| 68 | *Hystrix africaeaustralis* | SAf01 | South Africa | FJ472544 | 3 | 12S rRNA |
| 69 | *Hystrix africaeaustralis* | SAf04 | South Africa | FJ472545 | 3 | 12S rRNA |
| 70 | *Hystrix indica* | In02 | Israel | FJ472546 | 3 | 12S rRNA |
| 71 | *Hystrix cristata* | / | / | AY093659 | 3 | 12S rRNA |
| 72 | *Hystrix africaeaustralis* | / | / | U12448 | 3 | 12S rRNA |
